# Applying interpersonal neuroscience for understanding classroom learning in students with ADHD

**DOI:** 10.3389/fpsyg.2025.1690093

**Published:** 2025-12-17

**Authors:** Vanessa Reindl, Nastassja L. Fischer, Kerstin Konrad, Victoria Leong

**Affiliations:** 1 Child Neuropsychology Section, Department of Child and Adolescent Psychiatry, Psychosomatics and Psychotherapy, Uniklinik RWTH Aachen, Aachen, Germany; 2 Psychology, School of Social Sciences, Nanyang Technological University, Singapore, Singapore; 3 Centre for Research in Pedagogy and Practice (CRPP), National Institute of Education (NIE), Nanyang Technological University, Singapore, Singapore; 4 Science of Learning in Education Centre (SoLEC), National Institute of Education (NIE), Nanyang Technological University, Singapore, Singapore; 5 JARA-BRAIN Institute II, Molecular Neuroscience and Neuroimaging, RWTH Aachen & Research Centre Jülich, Jülich, Germany; 6 Early Mental Potential and Wellbeing Research (EMPOWER) Centre, Nanyang Technological University, Singapore, Singapore

**Keywords:** ADHD, learning, school, interpersonal synchrony, hyperscanning

## Abstract

Interpersonal neuroscience has gained importance in complementing single-person approaches to understand the neural underpinnings of learning in social contexts, mainly in neurotypical adults and children. This Perspective explores how such methods could be leveraged to improve our understanding of classroom learning in children with diverse educational needs, in particular with Attention Deficit/Hyperactivity Disorder, taking into account their unique symptomatology and the challenges they face in social and educational settings.

## 1 Introduction

Cognitive neuroscience has significantly advanced our understanding of the brain processes and regions involved in learning (e.g., Houdé et al., 2010). However, a limitation of these studies is the simplified and often artificial nature of the settings in which data is typically gathered, e.g., participants lie in a functional magnetic resonance imaging (fMRI) scanner or complete tasks alone in front of a computer. While these controlled conditions are essential for analyzing specific cognitive functions, they also create a significant gap between the experimental environment and the settings in which real-world learning occurs (Han et al., 2019). Classrooms are dynamic and socially complex settings where interactions among students and between students and teachers significantly influence both emotional and cognitive learning experiences (Reindl et al., 2024). Further, unlike laboratory environments, classrooms are rarely free from distractions; they are typically noisy, stimulus-rich, and unpredictable, with potential distractions arising both from peers and the surrounding environment. These conditions are especially relevant when considering students with diverse learning needs or neurodevelopmental disorders. For example, children with Attention Deficit/Hyperactivity Disorder (ADHD) more often experience difficulties in paying attention in a noisy and distracting classroom environment (Kofler et al., 2008; Stokes et al., 2022), or forming positive, collaborative relationships with their teachers (MacLean et al., 2023). Consequently, the brain measurements of these children, when collected in isolation, may fail to reflect deficits that emerge specifically in such complex, social interactive environments.

**Figure 1:**
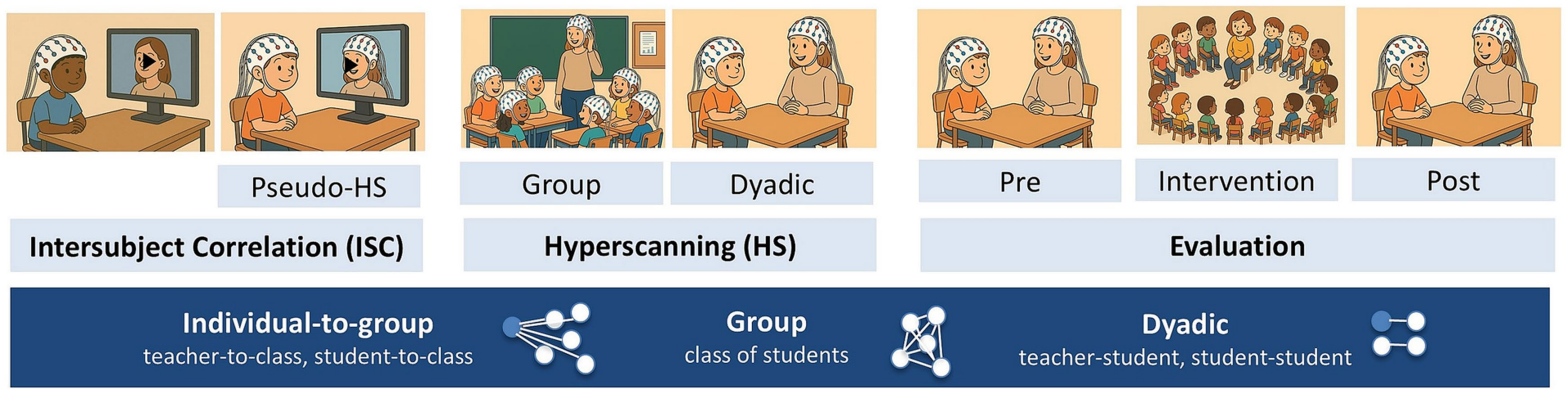
Experimental design. Intersubject correlation (ISC) studies record the brain activities of subjects individually to the same time-locked stimuli (e.g., movies) or sequentially, e.g., recording the brain activities of the teacher first and afterwards recording the brain activities of the student while watching a video of the teacher. This is also known as pseudo-hyperscanning. Hyperscanning studies measure the brain activities of multiple subjects concurrently during their real-time social interaction and can be applied in dyadic and in group settings. Both ISC and hyperscanning could serve as additional measures to evaluate the effectiveness of classroom-based interventions. Inter-brain synchrony (IBS) can be analyzed at multiple levels, including the individual-to-group, group, and dyadic level.

We suggest that recent advances in multi-person neuroscience may offer new possibilities for understanding the neural mechanisms underlying real-world classroom learning in diverse students and potentially informing tailored teaching strategies. A first step toward ecologically valid measurements is shifting from static and isolated stimuli to dynamic ones, like measuring brain activity during video lectures. Such intersubject correlation (ISC) studies assess the correlation between participants’ neural responses to shared stimuli, assuming that if participants watch the same videos or listen to narratives, brain responses that are systematically driven by the presented stimuli fluctuate synchronously across participants (Nastase et al., 2019). The ISC approach can be extended by first recording a teacher’s brain activity during a lecture, then recording students’ responses to that lecture to investigate teacher-to-learner brain synchronization (e.g., Nguyen et al., 2022). Additionally, measuring the brain activities of students and teacher simultaneously by using a hyperscanning paradigm would allow for exploring the dynamic, reciprocal teacher-student or student–student interactions during real-time classroom learning exchanges (e.g., Davidesco et al., 2023; see Figure 1; Supplementary Table 1 on key study characteristics and recommendations on how to design ISC / hyperscanning studies on learning).

Studies utilizing ISC and hyperscanning methods suggest that learning is supported by synchronization of brain signals between teachers and students, as well as among peers (e.g., Nguyen et al., 2022; Davidesco et al., 2023; Meshulam et al., 2021). This phenomenon, here collectively referred to as interbrain synchrony (IBS), may not only serve as a marker of learning (Zhang et al., 2022) but could also play an active role in facilitating learning (Pan et al., 2021). This facilitation could either occur directly, e.g., by enhancing perceptual encoding (Wass et al., 2020), though this has yet to be empirically demonstrated, or indirectly, e.g., via its association with motivational-emotional and social-cognitive alignment between learner and instructor (Reindl et al., 2024). While the role of IBS as a neurobiological marker of learning has been supported by a recent meta-analysis showing a significant positive correlation between IBS and learning outcomes across 16 studies (*r* = 0.444, *p* < 0.001, 95% CI [0.34, 0.54]; Zhang et al., 2022), the mechanistic contribution of IBS to learning remains largely speculative. Preliminary evidence suggests that applying synchronous transcranial alternating current stimulation (tACS) with the same frequency to two individuals, which is intended to enhance IBS, may facilitate learning, as demonstrated by improved intonation learning performance of a song (Pan et al., 2021) or greater conceptual alignment in a semiotic game (Chen et al., 2022). However, these findings require replication and extension to other forms of learning to establish the generalizability and causal nature of the observed effects, as well as the influence of potential mediators (e.g., movement synchrony (Pan et al., 2021)). IBS is also sensitive to contextual factors like teaching methods and instructions, being influenced by the quality of social interactions and closeness of social relationships (e.g., Dikker et al., 2017). A recent review indicated that an increasing number of hyperscanning studies assesses learning by recording IBS between instructor and learner dyads (*N* = 15 until March 2023), with only few being conducted in real classroom settings (of these, *N* = 3; Tan et al., 2023). Further, no hyperscanning study on learning that we know of has been conducted in children with diverse learning needs.

Thus, in this Perspective article, our aim is to explore how multi-person neuroscience approaches can deepen our understanding of classroom learning in children with diverse learning needs, specifically with ADHD. After reviewing the literature on academic challenges faced by children with ADHD (Section 2), we discuss how these methods could be leveraged to explore key aspects of learning, including how attention is maintained or lost during classroom activities (Section 3) and how students interact with teachers and peers (Section 4). Finally, we outline how IBS measurements could be integrated to design more effective interventions (Section 5) before critically discussing challenges and opportunities of such methods (Section 6).

It should be noted that while here we specifically focus on students with ADHD, many of the challenges discussed also apply to students in general and to other groups at risk of poorer academic outcomes. Teacher–student relationships, for example, are consistently linked to student achievement across populations, with stronger associations observed in samples with more students facing learning difficulties or with lower socio-economic status (Roorda et al., 2011). Similarly, research shows that boys from special education for Autism Spectrum Disorders (ASD) report higher levels of conflict and lower engagement in school compared to peers from regular education, with the level of conflict exerting a stronger negative influence on engagement for boys from special education (Roorda et al., 2021).

## 2 ADHD and academic challenges

Children diagnosed with ADHD are characterized by a persistent pattern of inattention and/or hyperactivity-impulsivity that interferes with their everyday functioning or development (DSM-5). Academic difficulties are prevalent across both genders and tend to be particularly pronounced in younger children compared to adolescents and adults (Frazier et al., 2007). In a large 8-year longitudinal study (*N* = 1,264), youths with high hyperactivity-inattention symptoms were two to three times more likely to experience negative academic outcomes, including grade retention, failure to graduate from secondary school, lower diploma attainment, and lower overall academic performance (Galéra et al., 2009). A comprehensive meta-analysis of 176 studies further confirmed the long-term academic impact of untreated ADHD symptoms, with 79% of the reported achievement test outcomes and 75% of academic performance outcomes being worse compared to non-ADHD controls (Arnold et al., 2020). Importantly, the meta-analysis also highlighted that appropriate interventions, especially multimodal treatments, can substantially improve academic outcomes.

The fundamental behavioral characteristics of ADHD, including deficits in executive function, may play a significant role in the academic challenges students face. For instance, inattention can lead to a lack of focus in the classroom, and an inability to follow instructions. Hyperactivity may lead to difficulty staying seated, excessive fidgeting and talking, which can cause teachers to view classroom behaviors negatively. Impulsivity may interfere with the ability of planning ahead of time, e.g., affecting performance on academic tests or long-term projects.

Etiological models of ADHD emphasize multiple causal pathways, involving both cognitive control deficits and delay aversion abilities (Sonuga-Barke, 2005). These are both associated with top-down control brain networks that undergo significant developmental changes during early childhood (Pauli-Pott et al., 2019). For example, individuals with ADHD often exhibit a preference for smaller, immediate rewards over larger, delayed ones, which can negatively impact academic success (Luman et al., 2010). This preference, often described as “delay aversion” or “temporal discounting”, can lead to difficulties in focusing on long-term goals and completing tasks that require sustained effort. Thus, motivational deficits are core features of the disorder and together with cognitive control impairments, they can negatively impact academic success (Smith and Langberg, 2018).

ADHD is also a highly heterogeneous condition with different presentations (according to DSM-5: predominantly inattentive, predominantly hyperactive–impulsive and combined), different symptom profiles and frequent comorbidities. Relatively few clinic-referred individuals are free of co-morbidity, while some patients have a complex pattern of multiple problems (Gnanavel et al., 2019; Faraone et al., 2024; Reale et al., 2017). These may include communication disorders, intellectual disabilities, sleep disorders, specific learning disabilities, mood disorders, oppositional defiant disorder (ODD) / conduct disorder (CD), anxiety disorders, tic disorders, ASD and substance use disorders (for an overview see Gnanavel et al., 2019; Faraone et al., 2024; Reale et al., 2017). Such comorbid disorders can affect classroom behavior and academic performance (e.g., Abikoff et al., 2002; Cuffe et al., 2020). For example, in a classroom behavioral observation study, children with ADHD and comorbid disruptive behavior disorders (ages 7–10 years) displayed higher rates of interference (e.g., clowning, interrupting others, talking during work), off-task (sustained inattention or distractibility) and aggressive behaviors in the classroom compared to their classmates with ADHD only or ADHD with comorbid anxiety disorders (Abikoff et al., 2002). Further, in a community-based sample, children with ADHD and comorbid anxiety or mood disorders (ages 4 to 15 years) had an increased risk of poor academic performance compared to those with ADHD only (odds ratio [OR] = 10.8; confidence interval [CI] = [2.4, 49.1]; Cuffe et al., 2020). Thus, despite the well-documented academic challenges associated with ADHD at group level, significant variability exists in individual symptomatology, impairment, and developmental trajectories, underscoring the importance of considering individual differences in developmental research.

## 3 IBS as an index of classroom engagement

Many studies have documented significant deficits in classroom attention in children with ADHD. A meta-analysis across 23 observational studies reported a large effect size (ES = 0.73) for between-group differences (ADHD vs. controls) which might even be an underestimation of the classroom visual attention deficits (Kofler et al., 2008). After accounting for confounding factors, children with ADHD were on-task approximately 75% of the time compared to 88% for their classroom peers (ES = 1.40; Kofler et al., 2008). Their attentive behavior was more variable (Kofler et al., 2008), and they struggled more often to return to task-relevant stimuli once distracted than typically developed participants (Stokes et al., 2022). Further evidence from using eye-tracking data collected in a virtual reality classroom demonstrated that even short distractions can disrupt task performance in children with ADHD (Stokes et al., 2022).

Shared attention has been proposed as a likely source of IBS in classrooms (Dikker et al., 2017). IBS may reflect students’ neural entrainment to common sensory inputs—such as the teacher, learning materials (e.g., videos), or peers (e.g., during group discussions). Evidence shows that electroencephalography (EEG) rhythms align with the temporal structure of auditory and audiovisual stimuli, which is amplified when these stimuli are attended to (Lakatos et al., 2008; Zion Golumbic et al., 2013). Therefore, individuals engaged with a same stimulus may exhibit a high IBS. Additional findings indicate that students’ ISCs during educational videos correlated with exam performance, regardless of whether students had prior knowledge of a post-test (intentional learning) or not (incidental learning; Cohen et al., 2018). ISCs were also higher in the intentional compared to the incidental learning condition, although no significant behavioral differences were found in test performance. This dichotomy may happen because neural measures may be more sensitive to subtle differences in student engagement than behavioral metrics. However, when students’ attention was divided (by silently counting backward from 1,000 in steps of seven), ISCs decreased significantly and were unrelated to exam performance (Cohen et al., 2018). These findings were confirmed by a recent meta-analysis revealing a strong positive relationship between ISC and attention (*r* = 0.65, *p* < 0.001; *N* = 14 studies and 27 effect sizes), demonstrating that ISC may serve as an effective neural marker for attentional engagement (Liu et al., 2025). Similarly, a hyperscanning study showed that decreased EEG alpha-band power, an indicator of increased attention, was linked to greater alpha-band IBS (coherence) between students and their group (Dikker et al., 2017).

When compared to healthy controls, adults with ADHD, showed reduced ISCs, measured by fMRI, in attention-related and sensory brain regions while they were engaged a film-based multi-talker scenario that included periods with and without background auditory distractors (Salmi et al., 2020). Specifically, ISCs in the posterior parietal cortex were weaker in adults with ADHD when irrelevant speech or music was being presented in the background, and regionally distinct ISC signatures emerged for inattention and impulsivity symptoms (Salmi et al., 2020).

**Figure 2:**
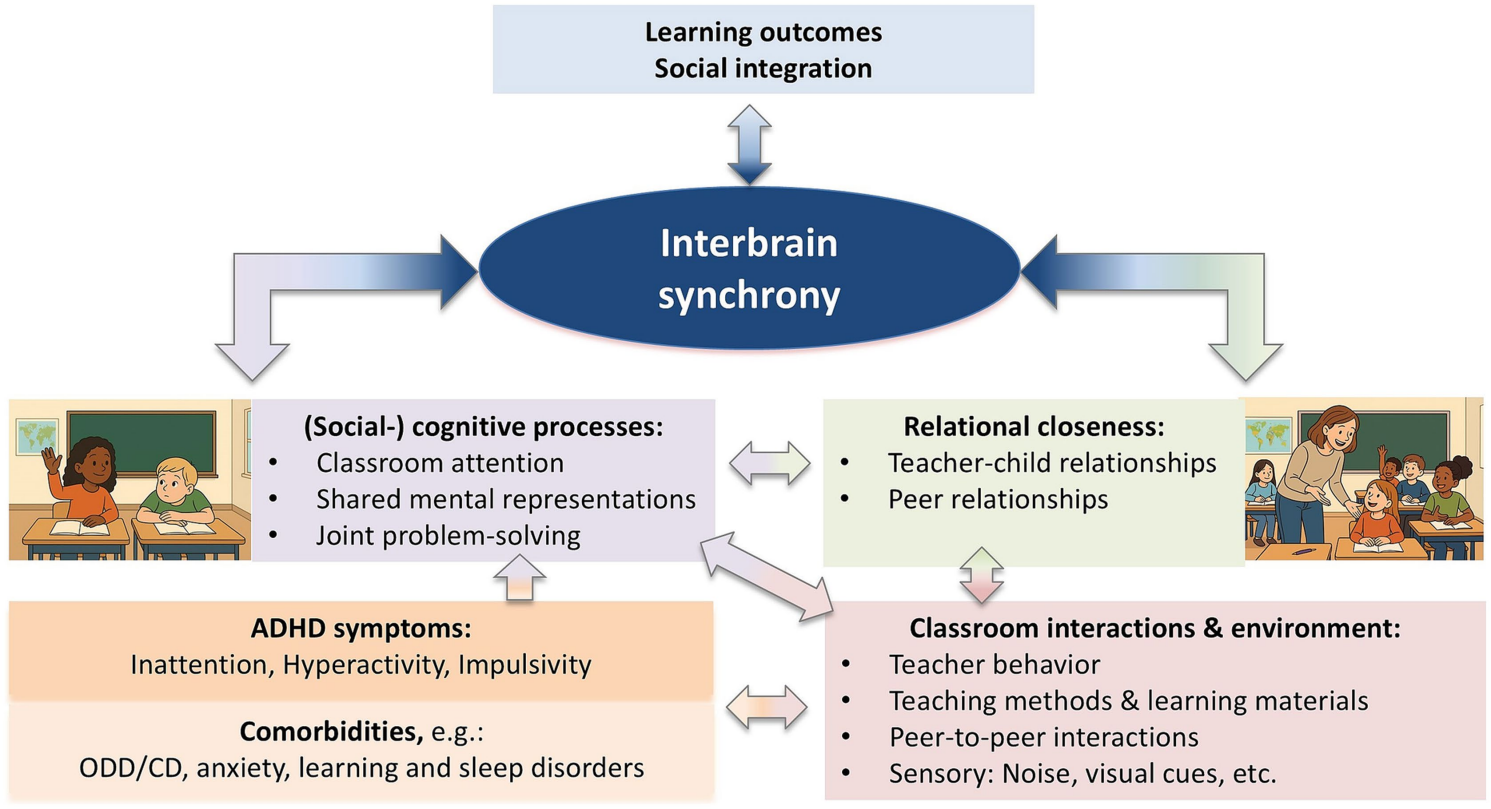
Proposed pathways of how IBS can serve as a marker and facilitator of classroom learning in students with ADHD. ADHD and its comorbid symptoms (e.g., anxiety disorders, oppositional defiant disorder (ODD)/conduct disorder (CD), learning disorders, and sleep disorders) can directly interfere with the (social-)cognitive processes involved in learning. These processes include sustaining attention, forming accurate mental representations of learned concepts, or engaging in collaborative learning activities, such as joint problem solving. In addition, ADHD and its comorbid symptoms may interact with features of the classroom environment (e.g., noise levels) and classroom interactions (e.g., teacher behavior, peer relationships), influencing both (social-)cognitive processes and relational closeness between student peers and between teacher and students. Both shared (social-)cognitive processes and relational closeness have been linked to inter-brain synchrony (IBS) in empirical studies. In turn, higher levels of IBS have been positively associated with social integration and learning outcomes. The model is adapted from Reindl et al. (2024) and extends it to children with ADHD.

In addition to ISC and IBS metrics, individual brain markers (e.g., EEG spectral power) can also provide insights into students’ engagement during classroom activities. For instance, during a sustained attention task in a classroom setting, prolonged response times were preceded by increased delta and theta power and decreased beta power over occipital and temporal regions (Ko et al., 2017). Furthermore, children’s alpha power was found to be highest during a mindfulness session compared to teacher-led and student-led classroom activities, indicating an internal attention focus during the activity (Xu et al., 2022). Although we need more evidence on the extent to which ISC, hyperscanning or other brain measures predict learning outcomes, these methods could possibly yield complimentary insights. Therefore, IBS, in combination with individual brain measurements, may help to track mutual attention in educational settings. For example, it could be used to understand how different teaching strategies and learning environments support children with ADHD in staying on task and re-engaging with the lesson after being distracted, as well as to identify distractors that can then be eliminated. A proposed model of how IBS measures are related to classroom learning in children with ADHD is depicted in Figure 2.

## 4 IBS to measure teacher-student and peer relationships

Teacher-student relationships are critical for learning success, yet students with ADHD symptoms often experience less close and more conflictual relationships with their teachers, which also agrees with teachers’ own perceptions (Ewe, 2019). A meta-analysis of 27 studies found that children with ADHD symptoms tended to have relationships with their teachers that were low in closeness (*r* = − 0.170) and high in conflict (*r* = 0.414; MacLean et al., 2023). Children with ADHD symptoms frequently perceive teachers as overly directive and report feeling less competent and connected in the classroom, regardless of age, co-occurring conduct problems or reading ability (Rogers and Tannock, 2018). Research on teacher-student “working alliance” further indicates that students with ADHD symptoms reported weaker emotional and collaborative bonds with their teachers, controlling for children’s conduct problems and academic achievement (Rogers et al., 2015). Longitudinal evidence supports the impact of early relational dynamics: negative teacher-student relationships in kindergarten have been shown to predict poorer academic and behavioral outcomes through eighth grade, particularly among boys and children with elevated behavior problems (as rated by the kindergarten teacher, comprising of conduct, learning and shy/anxious problems; Hamre and Pianta, 2001). Additionally, the presence of students with ADHD in classrooms has been associated with increased teacher stress and perceptions of environmental unpredictability (i.e., “entropy”; Fabio et al., 2023). It was found that higher numbers of students with ADHD in the classroom were linked to greater perceived disorder and elevated stress, with greater levels of personal entropy (i.e., a sense of internal chaos or disorganization) strongly predicting higher stress levels. This suggests that ADHD symptoms in students can be perceived as disruptive to the classroom dynamics and teacher well-being, pointing to the need for targeted teacher training. Beyond teacher relationships, students with ADHD more often face additional difficulties with peers, including stigmatization, rejection and victimization, which are associated with negative long-term outcomes (Gardner and Gerdes, 2015).

An increasing number of neuroimaging studies suggest that the quality of social interactions and relationship closeness are associated with variations in IBS (e.g., Dikker et al., 2017; Zheng et al., 2020; Bevilacqua et al., 2019). An EEG hyperscanning study found that pairs of students who engaged in a two-minute silent eye contact prior to class had a higher IBS during class, and this correlated with students’ mutual closeness ratings, demonstrating that IBS can be modulated by classroom social dynamics (Dikker et al., 2017). Similarly, student-teacher closeness ratings have been found to predict student-teacher IBS during lectures, but not during the viewing of videos (Bevilacqua et al., 2019). This aligns with studies performed with participants forming close relationships (e.g., parent–child or romantic partners), where increased IBS is present during interactions, particularly in frontal, temporal, and parietal regions (Zhao et al., 2024). Recent theories propose that such increases in IBS may result from interbrain plasticity, whereby repeated social interactions induce experience-dependent short- and long-term changes in neural coupling between interaction partners (Shamay-Tsoory, 2022; Shamay-Tsoory et al., 2024).

Very few studies have investigated interpersonal synchrony in children or adults with ADHD. At the behavioral level, preschoolers with elevated ADHD symptoms exhibited lower mother–child synchrony, measured via affective mutuality, mutual enjoyment, and reciprocal interactions during free and structured play, which accounted for unique variance in children’s functioning, beyond the effects of ADHD symptom severity and IQ (Healey et al., 2010). In adults with ADHD, impaired intentional (but not spontaneous) interpersonal behavioral synchronization was observed during an interactive task, suggesting that ADHD may selectively impair cognitive control mechanisms required for voluntary synchronizing efforts (Gvirts Problovski et al., 2021). At the neural level, an EEG-based ISC study found that children aged 6–14 years with ADHD showed altered brain synchronization while watching basketball videos varying in levels of verbal and non-verbal social interaction (Yang et al., 2024). Compared to their typically developing peers, children with ADHD exhibited a significantly smaller difference in synchronized gamma oscillations between high and low social interaction videos, indicating distinct spatiotemporal neural responses associated with social information processing in ADHD.

Therefore, albeit limited current evidence, IBS may prove to be a sensitive metric to capture affective qualities of student-teacher and student–student interactions, providing information about student engagement and class dynamics beyond teacher- or student-reports or classroom observations.

## 5 IBS to design classroom-based interventions

Both pharmacological and behavioral interventions have been shown to improve ADHD symptoms and functional outcomes (see NICE guidelines, AACAP Practice Parameters). Classroom-based behavioral interventions (together with parent trainings) are considered well-established, effective non-pharmacological interventions that have been shown to improve children’s academic outcomes, attention and organizational skills as well as reduce their externalizing behaviors in the classroom (DuPaul and Eckert, 1997, Evans et al., 2018, 2014).

For example, the *Making Socially Accepting Inclusive Classrooms* (MOSAIC) program provides teachers with strategies to manage behavioral problems and foster peer inclusiveness, thereby promoting a positive classroom climate (Mikami et al., 2022). Compared to a control group, children in MOSAIC classrooms, both with and without elevated ADHD symptoms, were rated by teachers as having higher social and academic competencies and fewer ADHD-related impairments by the end of the school year (*N* = 34 teachers; *N* = 558 children; mean age = 7 years; Mikami et al., 2022). However, while target children (those with elevated ADHD symptoms and peer impairment) in MOSAIC classrooms reported more supportive teacher relationships, were also rated less favorably by peers on sociometric measures than target children in control classrooms. This underscores the persistent challenge of shifting peer perceptions and highlights the need for interventions that address both teacher-student and peer dynamics.

Incorporating IBS metrics into the evaluation of such interventions may offer a more objective assessment of their effectiveness, particularly regarding relational dynamics. Notably, the majority (i.e., 75%) of the studies published up to 2022 investigating the effectiveness of interventions on teacher-student relationships used only teacher reports as the outcome measure (Poling et al., 2022). Given the dyadic nature of this phenomenon, solely relying on one informant may hinder the understanding of the true real-world effects of such interventions and may increase the risk of biases. Therefore, we suggest that IBS could be an additional, objective tool to assess the effects of intervention programs or specific strategies on classroom relationships (including a measure from teacher and student). For instance, dyadic teacher–student hyperscanning could be conducted before and after an intervention to assess changes in IBS and to determine whether improvements in reported relational quality are paralleled by changes in IBS, offering objective, convergent evidence of intervention effectiveness and deepening our understanding of the underlying mechanisms.

Further, hyperscanning studies have shown that IBS is sensitive to contextual factors such as teaching mode (e.g., lecture, video, or group discussion (Dikker et al., 2017)) and instructional techniques (e.g., Pan et al., 2020). Although some studies found no association between IBS and learning outcomes (Bevilacqua et al., 2019), others reported associations only under specific conditions, such as during scaffolding rather than explanation-based learning (Pan et al., 2020), interactive (with turn-taking) over noninteractive learning (without turn-taking; Pan et al., 2018) and elaborated versus simple feedback (Zhu et al., 2022). These findings indicate that IBS may serve as a useful neural marker for understanding how different teaching approaches promote cognitive alignment between teachers and students. In turn, this could help to tailor interventions, e.g., with respect to instructional or task modifications, to better meet the learning needs of students with ADHD.

Finally, if the link between IBS and learning outcomes is further substantiated, this will also allow to develop ‘*synchrony-informed’* classroom interventions. For example, teachers could be trained to use eye contact, joint attention cues (e.g., pointing, nodding), to be attentive to students’ emotional and behavioral cues and adjust their teaching behaviors accordingly. In a classroom study, students with ADHD could be randomly assigned to either a synchrony-enhancing instructional condition (with training in these behaviors) or a standard instruction condition. Hyperscanning could measure IBS during both conditions, while behavioral measures track task engagement and comprehension. If the synchrony-enhancing condition leads to increased IBS and better engagement learning outcomes, it would support the idea that modulating interactional features can directly improve learning outcomes in students with ADHD.

## 6 Challenges and opportunities

Although promising, incorporating IBS metrics in studies with children with diverse learning needs still comes with significant limitations. The concept of IBS as an index of social attunement is still under debate, with some researchers arguing that apart from the lack of a clearer definition of what different studies mean by interpersonal or interbrain synchronization, “true” interneural synchronization would be different from neural entrainment, motor-induced neural synchrony, or attention-enhanced neural synchrony (Holroyd, 2022). Accordingly, stronger causal evidence (e.g., using multi-brain stimulation, Pan et al., 2021; Chen et al., 2022; Novembre and Iannetti, 2021) is needed to show that IBS does indeed make a mechanistic contribution to social interactions, and consequently to social aspects of learning and cognition. With respect to neuroimaging research in children with ADHD, there are additional challenges that need to be considered. Hyperactivity and impulsivity can cause increased or atypical movement during recordings, leading to more and varied signal artifacts that may confound group comparisons. Additionally, psychostimulant medication taken during class can affect brain signals. Although these challenges can be partially addressed through advanced motion correction techniques or by conducting sessions when children are medication-free, e.g., on weekends or during holiday, the high variability in symptoms and frequent comorbidities remain significant obstacles. For example, several studies have reported negative associations between ASD symptom severity and social difficulties on the one hand, and IBS on the other hand (e.g., Key et al., 2022; Quiñones-Camacho et al., 2021), although also inconsistent findings have been noted (e.g., Kruppa et al., 2021; see Li et al., 2025 for a recent meta-analysis). While an increasing number of hyperscanning studies has explored IBS in participants with ASD (*N* = 8 studies published until 2024, Li et al., 2025), for other comorbid disorders, the effects on IBS remain largely unexplored, complicating the interpretation of findings. One promising strategy to address this complexity may be adopting a transdiagnostic, dimensional approach (Musser and Raiker, 2019).

Finally, employing portable and wearable brain technologies in real classroom settings presents significant technical, methodological, and ethical challenges (for a review, see Nouri, 2025). From a technical and methodological perspective, the high costs of many neuroimaging systems and the need for intensive researcher involvement often limit the number of neuroimaging devices available and number of participants that can be recorded, while more affordable systems tend to offer fewer recording channels, reducing spatial coverage and possibly data quality. Additionally, conducting recordings in classrooms entails less stringent experimental control compared to laboratory settings and is more susceptible to background noise, which may further reduce data quality. From an ethical perspective, implementing hyperscanning in the classroom could interfere with normal classroom routines and raise issues related to privacy and informed consent. The acceptability of neuroimaging technologies among teachers and students is another critical consideration, and particularly, students who choose not to participate must be appropriately accommodated. Yet, enhancing ecological validity does not necessarily require data collection in real classrooms. The use of naturalistic stimuli, such as video-based lectures, and the integration of immersive technologies (e.g., virtual, augmented, or mixed reality) could for instance help to investigate how attention is maintained or lost during instruction and allow for experimentally controlled manipulations of distractors. Moreover, learning, social and affective processes could also be effectively examined in smaller, controlled environments such as dyadic teacher–student interactions or small-group settings in the lab, which may offer a balance between experimental control and ecological validity.

When these challenges are adequately addressed, capturing the dynamic neural interactions between individuals may offer novel insights into how teacher-student and peer dynamics influence students’ attention, motivation and behavior. Future research may aim to refine hyperscanning methodologies for use in educational environments, explore their potential as biomarkers for evaluating interventions or guiding teacher training, and identify new targets for tailored interventions.

## Data Availability

The original contributions presented in the study are included in the article/supplementary material, further inquiries can be directed to the corresponding authors.
