# Supplementary material for: Applying interpersonal neuroscience for understanding classroom learning in students with ADHD
Source: Front Psychol. 2025 Dec 17;16:1690093. doi: 10.3389/fpsyg.2025.1690093 (PMC12754603; doi:10.3389/fpsyg.2025.1690093)
Supplement: Supplementary file 1 [file Supplementary_file_1.pdf]

Supplementary Material

1 Supplementary Tables

Table 1: How to design Intersubject Correlation (ISC) studies and hyperscanning studies to measure learning.

Study Characteristics	Intersubject Correlation (ISC) studies	Hyperscanning
Setting	Subjects' brain activities are measured individually in response to the same time-locked stimuli, e.g., movies clips. Suited for learning formats with low level or no live social interaction (e.g., videos, lectures, online learning).	Brain activities of two or more subjects are measured at the same time during their interaction. Suited for learning formats with low to high level of social interaction (e.g., group discussion) and in a variety of settings: laboratory, real classrooms, dyadic or group settings, remote or face-to-face learning.
Neuroimaging modality	The choice of the most appropriate neuroimaging modality depends on the spatial and temporal scales relevant to the phenomenon, experimental design consideration and participant characteristics. Notably, portable electroencephalography (EEG) and functional near-infrared spectroscopy (fNIRS) devices can be used to measure brain activities in real-world environments (e.g., (1)). Furthermore, multimodal recordings combining different neuroimaging modalities (e.g., functional magnetic resonance imaging (fMRI)-EEG) or combining those with related measures (e.g., eye tracking or electrocardiography) can yield complementary temporal and spatial information and provide a more comprehensive understanding of the factors related to teacher-student / student-student IBS (e.g., (2)).	
Participants	Often a larger number of students or learners and a smaller number of teachers or experts is included. In some studies, the teacher is one of the experimenters and is kept constant across participants (e.g., (3)). While this may allow for greater standardization, using several teachers, either 'actual' teachers, 'lay' participants or trained researchers, introduces more variability and enhances generalizability across teachers. Additionally, student group size can affect group dynamics.	

Sample size	To determine the required sample size, an a priori power analysis can be conducted using effect sizes from prior studies, meta-analyses or theory. The meta-analysis by (4) reported an $r = 0.444$ (95% CI [0.34, 0.54], $p<0.001$) for the association between IBS and learning. Using this as an effect size, for a two-tailed Pearson correlation with $\alpha = 0.05$ and power = 0.80, the estimated minimum sample size would be N = 37 (analysis performed in G*Power3.1.9.7).	
Stimulus / task	IBS can be measured during learning, e.g., while watching / listening to lectures, after learning took place, e.g., watching segments from the lecture or recap videos, or during examination, e.g., answering open-answer questions (see (5,6)). The optimal stimulus duration depends on the time over which the stimulus conveys meaningful information, the signal frequencies of interest and the sampling rate of the device (7). In previous ISC studies on learning, stimulus durations varied, e.g., 57 – 215 s for video clips (EEG, (8)), up to 90 s for open answer questions (fMRI, (6)) or ~40-min lecture videos (in 3 - 5 segments; fMRI, (6)).	IBS is typically measured during learning but could also be assessed e.g., during oral examination. Analogous to ISC studies, the optimal duration of the task depends on the cognitive or behavioral processes involved, signal frequencies of interest and sampling rate. In previous hyperscanning studies on learning, task block durations varied, e.g., 30 s (teaching a video game repeated 6 times using fNIRS, (9)), 2 min. (videos repeated over 11 sessions using EEG, (1)) or 13 – 26 min. (teaching in turn-taking, lecturing or video mode using fNIRS, (10,11)).
Interbrain synchrony (IBS) measures	IBS quantifies statistical dependencies between neural signals from multiple participants using functional connectivity metrics whereby the appropriateness of certain estimator classes varies based on signal characteristics and study design. Metrics are classified as directed or non-directed, and as model-based or model-free (12). Non-directed metrics assess concurrent changes without considering any direction of influence, while directed metrics seek to establish a statistical causation from the data that is based on the principle that cause precedes effect. Model-based methods assume linearity; model-free ones capture non-linear interactions. Connectivity metrics can be computed from the time or frequency representation of a signal.	
Metrics	When there is no interaction between participants, e.g., when watching videos, only non-directional IBS metrics are typically used. Directional metrics	Directional and non-directional effects can be quantified.

		may provide additional insights when brain activities are recorded sequentially, e.g., of the student in response to pre-recorded stimuli of the teacher, by uncovering the delay by which information is transmitted from sender to receiver.	
	Levels of analysis	IBS can be calculated between pairs of subjects (e.g., teacher-to-student, student-to-student), between a group of subjects (e.g., class), or between individual subjects and a group of subjects (e.g., student-to-class). In ISC studies, it is mostly calculated between individual subjects and the group.	
	Determining significance	IBS metrics are affected by noise and should not be interpreted in absolute terms. Statistical significance is assessed via resampling (e.g., permutation or surrogate tests), condition contrasts (e.g., compared to baseline), or correlations with behavior/learning outcomes.	
Learning measures		Learning can be assessed using (pre- vs.) post-test scores or other behavioral assessments, e.g., performance ratings by experts. A pre- vs. post-test design allows to account for different baseline levels and to identify participants with near-perfect pre-test scores. Learning can be assessed using various types of achievement test items, e.g., multiple-choice or essay items (see (13) for their respective advantages and disadvantages), and at different latencies, e.g., immediately after stimulus administration or several weeks later, akin to school exams or semester-long courses.	

References

1. Dikker S, Wan L, Davidesco I, Kaggen L, Oostrik M, McClintock J, et al. Brain-to-brain synchrony tracks real-world dynamic group interactions in the classroom. Curr Biol. 2017 May;27(9):1375–80.

2. Reindl V, Wass S, Leong V, Scharke W, Wistuba S, Wirth CL, et al. Multimodal hyperscanning reveals that synchrony of body and mind are distinct in mother-child dyads. NeuroImage. 2022 May;251:118982.

3. Piazza EA, Cohen A, Trach J, Lew-Williams C. Neural synchrony predicts children's learning of novel words. Cognition. 2021 Sept;214:104752.

4. Zhang L, Xu X, Li Z, Chen L, Feng L. Interpersonal neural synchronization predicting learning outcomes from teaching-learning interaction: a meta-analysis. Front Psychol. 2022 Feb 28;13:835147.

5. Nguyen M, Chang A, Micciche E, Meshulam M, Nastase SA, Hasson U. Teacher–student neural coupling during teaching and learning. Soc Cogn Affect Neurosci. 2022 Apr 1;17(4):367–76.

6. Meshulam M, Hasenfratz L, Hillman H, Liu YF, Nguyen M, Norman KA, et al. Neural alignment predicts learning outcomes in students taking an introduction to computer science course. Nat Commun. 2021 Mar 26;12(1):1922.

7. Nastase SA, Gazzola V, Hasson U, Keysers C. Measuring shared responses across subjects using intersubject correlation. Soc Cogn Affect Neurosci. 2019 Aug 7;14(6):667–85.

8. Zhu Y, Pan Y, Hu Y. Learning desire Is predicted by similar neural processing of naturalistic educational materials. eNeuro. 2019 Sept;6(5):ENEURO.0083-19.2019.

9. Takeuchi N, Mori T, Suzukamo Y, Izumi SI. Integration of teaching processes and learning assessment in the prefrontal cortex during a video game teaching–learning task. Front Psychol. 2017 Jan; 7.

10. Zheng L, Liu W, Long Y, Zhai Y, Zhao H, Bai X, et al. Affiliative bonding between teachers and students through interpersonal synchronisation in brain activity. Soc Cogn Affect Neurosci. 2020 Jan 30;15(1):97–109.

11. Zheng L, Chen C, Liu W, Long Y, Zhao H, Bai X, et al. Enhancement of teaching outcome through neural prediction of the students' knowledge state. Hum Brain Mapp. 2018 July;39(7):3046–57.

12. Bastos AM, Schoffelen JM. A tutorial review of functional connectivity analysis methods and their interpretational pitfalls. Front Syst Neurosci. 2016 Jan; 9.

13. Piontek ME. Best practices for designing and grading exams. Occas Pap. 2008;24:1–12.
